# Time to appropriate antimicrobial therapy serves an independent prognostic indicator in children with nosocomial *Klebsiella pneumoniae* bloodstream infection

**DOI:** 10.1186/s12887-022-03622-6

**Published:** 2022-10-03

**Authors:** Jie Cheng, Qinyuan Li, Guangli Zhang, Huiting Xu, Yuanyuan Li, Xiaoyin Tian, Dapeng Chen, Zhengxiu Luo

**Affiliations:** 1 Department of Emergency, Children’s Hospital of Chongqing Medical University, Chongqing Key Laboratory of Pediatrics, National Clinical Research Center for Child Health and Disorder, Ministry of Education Key Laboratory of Child Development and Disorders, Chongqing, 401122 China; 2 Department of Respiratory Medicine, Children’s Hospital of Chongqing Medical University, Chongqing Key Laboratory of Pediatrics, National Clinical Research Center for Child Health and Disorder, Ministry of Education Key Laboratory of Child Development and Disorders, Chongqing, 401122 China; 3 Department of Clinical Laboratory Center, Children’s Hospital of Chongqing Medical University, Chongqing, 401122 China

**Keywords:** *Klebsiella pneumonia*e, Delayed therapy, Time to appropriate therapy, Nosocomial bloodstream infection, Children

## Abstract

We tend to investigate the connection between time to appropriate therapy (TTAT) and prognosis in pediatric patients with nosocomial *Klebsiella pneumoniae* (*K. pneumoniae*) bloodstream infection, and find the optimal cutoff point for the empirical administration of antimicrobials. This retrospective study was conducted in Children’s Hospital of Chongqing Medical University, and inpatients with nosocomial *K. pneumoniae* bloodstream infection were finally enrolled. We applied the Classification and Regression Tree (CART) analysis to find the TTAT cutoff point and the Logistic Regression analysis to evaluate prognostic indicators. The incidence of septic shock and mortality was 17.91% (12/67) and 13.43% (9/67), respectively. The CART-derived TTAT cutoff point was 10.7 h. The multivariate logistic regression analysis indicated delayed therapy (TTAT ≥ 10.7 h), pediatric risk of mortality (PRISM) III scores ≥ 10, time to positivity (TTP) ≤ 13 h, and requiring for invasive mechanical ventilation were independently associated with the incidence of septic shock (Odds ratio [OR] 9.87, 95% Confidence interval [CI] 1.46–66.59, *P* = 0.019; OR 9.69, 95% CI 1.15–81.39, *P* = 0.036; OR 8.28, 95% CI 1.37–50.10, *P* = 0.021; OR 6.52, 95% CI 1.08–39.51, *P* = 0.042; respectively) and in-hospital mortality (OR 22.19, 95% CI 1.25–393.94, *P* = 0.035; OR 40.06, 95% CI 2.32–691.35, *P* = 0.011; OR 22.60, 95% CI 1.78–287.27, *P* = 0.016; OR 12.21, 95% CI 1.06–140.67, *P* = 0.045; respectively).

**Conclusions:** TTAT is an independent predictor of poor outcomes in children with nosocomial *K. pneumoniae* bloodstream infection. Initial appropriate antimicrobial therapy should be administrated timely and within 10.7 h from the onset of bloodstream infection is recommended.

## Introduction

*Klebsiella pneumoniae* (*K. pneumoniae*) is the most common antimicrobial-resistant gram-negative pathogens in nosocomial bloodstream infection, causing high economic burden [1]. Timely antimicrobial therapy is critical to the prognosis in patients with bloodstream infection [2]. According to the 2021 Surviving Sepsis Campaign [3], antimicrobials are recommended as soon as possible for sepsis patients (≤ 3 h for patients without shock, ≤ 1 h for patients with suspicious septic shock). The 1-h and 3-h goals are strongly recommended, while with low quality of evidence and remains controversial [3, 4]. Meanwhile, our previous study showed that the delayed appropriate antimicrobial therapy ≥ 13.6 h, not ≥ 1 or 3 h, was associated with the highest sepsis-related mortality in children with *Streptococcus pneumoniae* sepsis [5]. Furthermore, 1-h or 3-h goal sometimes is unrealistic to be achieved due to limitations in early recognition or diagnosis of sepsis [4]. In some ways, immediate antimicrobial treatment is lifesaving. However, overdiagnosis of sepsis and premature administration of antimicrobials may result in overtreatment and antimicrobial-associated harms [6, 7]. The Infectious Diseases Society of America states the administration time of antimicrobials vary with different pathogens and populations [8]. In adult patients, the optimal appropriate antimicrobial therapy time windows were 24 h for *K. pneumoniae* bloodstream infection [9], 48.1 h for *Enterococci* bloodstream infection [10], 52 h for *Pseudomonas aeruginosa* bloodstream infection [11] and 44.75 h for *Staphylococcus aureus* bacteremia [12]. Bacteremia patients with different pathogens could have different appropriate antimicrobial time windows and this remains unclear in pediatric patients with *K. pneumoniae* bloodstream. Therefore, the optimal antimicrobials administration time windows in different populations need to be explored. We tend to prove the prognostic value of time to appropriate therapy (TTAT) in children with nosocomial *K. pneumoniae* bloodstream infection, and to find an optimal time point for the appropriate antimicrobials administration.

## Methods

### Study designs and patients

This retrospective, observational cohort study was conducted in Children’s Hospital of Chongqing Medical University, National Clinical Research Center for Child Health and Disorder, ranked the top two children’s hospitals in China (rank list: http://top100.imicams.ac.cn/home). Patients diagnosed with *K. pneumoniae* bloodstream infection were enrolled. Inclusion was marked as follows: (i) inpatients, (ii) 1 month ≤ age ≤ 18 years, (iii) with monomicrobial *K. pneumoniae* bloodstream infection. The exclusion criteria were as the following: (i) patients diagnosed with community-acquired *K. pneumoniae* bloodstream infection, (ii) patients with incomplete clinical information and (iii) patients received appropriate antimicrobials against *K. pneumoniae* prior to blood culture. This is a retrospective study so that informed consent was exempted.

### Data collection and definitions

We retrospectively gathered the basic information (eg. Sex, weight and so on), underlying conditions, axillary temperature, sources of infection, microbiological and laboratory data, treatment and outcomes. Nosocomial infection was the infections occurred > 48 h after admission [13]. *K. pneumoniae* isolated from blood culture associate with related clinical manifestations of infection was diagnosed as *K. pneumoniae* bloodstream infection [13]. Patients with immunosuppression were characterized as patients with immunodeficiency diseases, or patients received chemotherapy or immunosuppressive steroid therapy more than 14 days [5]. Hypoalbuminemia was defined as intravascular albumin level < 2.5 g/dL for children younger than 7 months and < 3.4 g/dL for children 7 months or older [14]. Source of infection was defined according to the CDC /NHSN surveillance guidelines [15]. Disease severity of patients in different subgroups were compared by using the Pediatric Risk of Mortality (PRISM) III score [16]. Time to positivity (TTP) was characterized as the time interval from the start of incubation to the alert of bacterial growth [17]. Our previous study, for children with *K. pneumoniae* bloodstream infection, showed that TTP ≤ 13 h and a PRISM III score ≥ 10 were related to poor outcomes [18]. Empiric antimicrobial treatment was characterized as antimicrobials initially administrated without in vitro sensitivity test results [19]. Appropriate antimicrobial therapy was defined as patients received at least one intravenous antimicrobials documented in vitro susceptibility basing on the breakpoint established according to the Clinical and Laboratory Standards Institute (CLSI) guideline [20]. Multi-drug resistant (MDR) was defined as bacteria with resistance to 3 or more antimicrobials classes [21]. TTAT was defined as the time span between onset of bloodstream infection and the first dose of appropriate antimicrobials [9]. The onset of bloodstream infection was identified by no less than two senior infectious disease physicians according to clinical manifestations (e. g. fever, chill and so on) and biomarkers (e. g. C-reactive protein, procalcitonin and so on), and approved by the subsequent positive blood culture result. Sepsis and septic shock were diagnosed basing on the Sepsis-3.0 [22].

### Clinical outcomes

The primary outcome was in-hospital mortality, the second outcome was incidence of septic shock.

### Statistical analysis

Classification and regression tree (CART) analysis [23] was used to find the optimal cutoff point of TTAT and the area under the receiver operating characteristic (ROC) curve [24] was used to examine the prognostic value of the TTAT cutoff point. Kaplan–Meier survival analysis were used to compare the incidences of septic shock and mortality between early and delayed therapy groups which grouped according to the TTAT cutoff point. In-hospital mortality of different delay time spans to appropriate antimicrobials therapy were assessed by using linear χ^2^ test. In groups comparing, we applied the Manne-Whitney U test or the Student’s t test for inferential statistics of continuous data, and the Pearson χ^2^ test or the Fisher’s exact test for categorical variables. Logistic regression test was applied to find independent risk factors of poor outcomes. All variables were analyzed in multivariate analysis except for those with *P*-level ≥ 0.10 in univariate analysis, by using forward likelihood ratio selection. Odds ratio (OR) and the corresponding 95% confidence interval (CI) were calculated. All statistical analyses were conducted by SPSS software 23.0 for Windows. The level of significance was set at *P*-value < 0.05 (two-sided).

## Results

### Study population

**Fig. 1:**
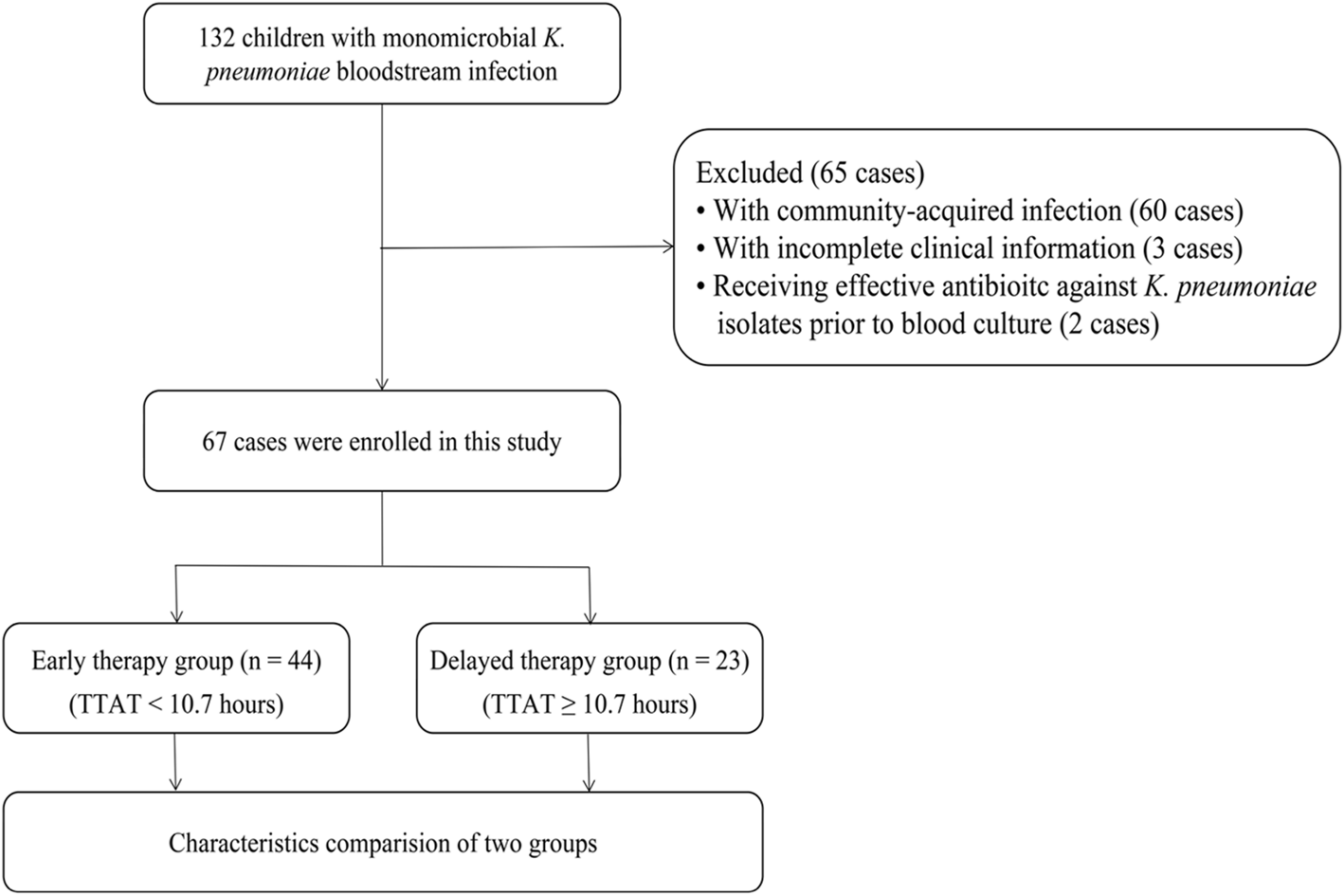
Flow diagram of the population

One hundred and thirty-two patients were retrospectively enrolled at the beginning. There were sixty-five patients were excluded: sixty cases were classified as community-acquired infection, three cases with incomplete clinical information, and two cases received effective antimicrobials against *K. pneumoniae* isolates prior to blood culture. Finally, sixty-seven cases were enrolled in this study (Fig. 1).

### Clinical characteristic of *K. pneumoniae* bloodstream infection in children

The median age was 4.33 (Inter-quartile range [IQR] 0.76–10.67) years, and the male accounted for 61.69% (42/67). More than half of the patients had hematologic malignancy or immunosuppression (44/67, 65.67%; 41/67, 61.19%, respectively). The most common source of bloodstream infections originated from respiratory tract (55.22%), followed by gastrointestinal tract (20.90%) and unknown source (14.93%). There were 32 (47.76%) extended-spectrum beta-lactamase (ESBL) positive and 6 (8.96%) multidrug resistant (MDR) *K. pneumoniae* isolates. More than half of the *K. pneumoniae* isolates resistant to sulbactam (40/67, 59.70%). The resistant rates of cephalosporin and tazobactam were 22.39% (15/67) and 20.90% (14/67), respectively. There were 28 (41.79%) patients received antimicrobial therapy prior to blood culture. Furthermore, thirty-eight (56.72%) patients were treated with carbapenems empirically before the susceptibility tests. The median TTP and TTAT was 14.12 (IQR 12.72–16.22) hours and 4.52 (IQR 0.97–14.18) hours, respectively. Twenty-two (32.84%) patients with secondary hypoalbuminemia and eleven (16.42%) patients administered with invasive mechanical ventilation during hospitalization. The median length of stay before the onset of bloodstream infection was13.68 (IQR 6.59–17.53) days, the median length of whole hospitalization stay was 28.96 (IQR 20.04–42.75) days. Septic shock occurred in 17.91% (12/67) of patients. The in-hospital mortality was 13.43% (9/67). The detailed characteristics of those patients are presented in Table 1.

**Table 1:** Clinical characteristics of 67 children with nosocomial *K. pneumoniae* bloodstream infection

Characteristics	Number/median	Percent/IQR
Demographic characteristics		
Male (n, %)	42	61.69
Age (years) (median, IQR)	4.33	0.76–10.67
Underlying conditions		
Hematologic malignancy (n, %)	44	65.67
Immunosuppression (n, %)	41	61.19
Congenital heart disease (n, %)	14	20.90
Sources of infection		
Respiratory tract (n, %)	37	55.22
Gastrointestinal tract (n, %)	14	20.90
Unknown source (n, %)	10	14.93
Invasive operation (n, %)	5	7.46
Urinary tract (n, %)	1	1.49
Drug resistant bacteria phenotypes		
Sulbactam resistant (n, %)	40	59.70
Extended spectrum beta-lactamase (n, %)	32	47.76
Cephalosporin resistant (n, %)	15	22.39
Tazobactam resistant (n, %)	14	20.90
Carbapenem resistant (n, %)	7	10.45
Multidrug resistant (n, %)	6	8.96
Aminoglycoside resistant (n, %)	4	5.97
Empiric antimicrobial treatment		
Carbapenem (n, %)	38	56.72
Fourth-generation cephalosporin (n, %)	9	13.43
Third-generation cephalosporin (n, %)	8	11.94
Tazobactam (n, %)	7	10.45
Second-generation cephalosporin (n, %)	3	4.48
Sulbactam (n, %)	2	2.99
Length of stay before the onset of bloodstream infection (days) (median, IQR)	13.68	6.59–17.53
Length of hospitalization stay (days) (median, IQR)	28.96	20.04–42.75
The peak of temperature (centigrade) (median, IQR)	39.8	39.3–40.1
Antimicrobials administrated prior to blood culture (n, %)	28	41.79
With secondary hypoalbuminemia during hospitalization (n, %)	22	32.84
PRISM III score (median, IQR)	8	3–9
TTP (h) (median, IQR)	14.12	12.72–16.22
TTAT (h) (median, IQR)	4.52	0.97–14.18
Need for invasive mechanical ventilation (n, %)	11	16.42
Septic shock (n, %)	12	17.91
In-hospital mortality (n, %)	9	13.43

*Abbreviations*: *IQR* inter-quartile range, *PRISM* pediatric risk of mortality, *TTAT* time to appropriate therapy, *TTP* time to positivity

### TTAT of *K. pneumoniae bloodstream* infection in children

**Fig. 2:**
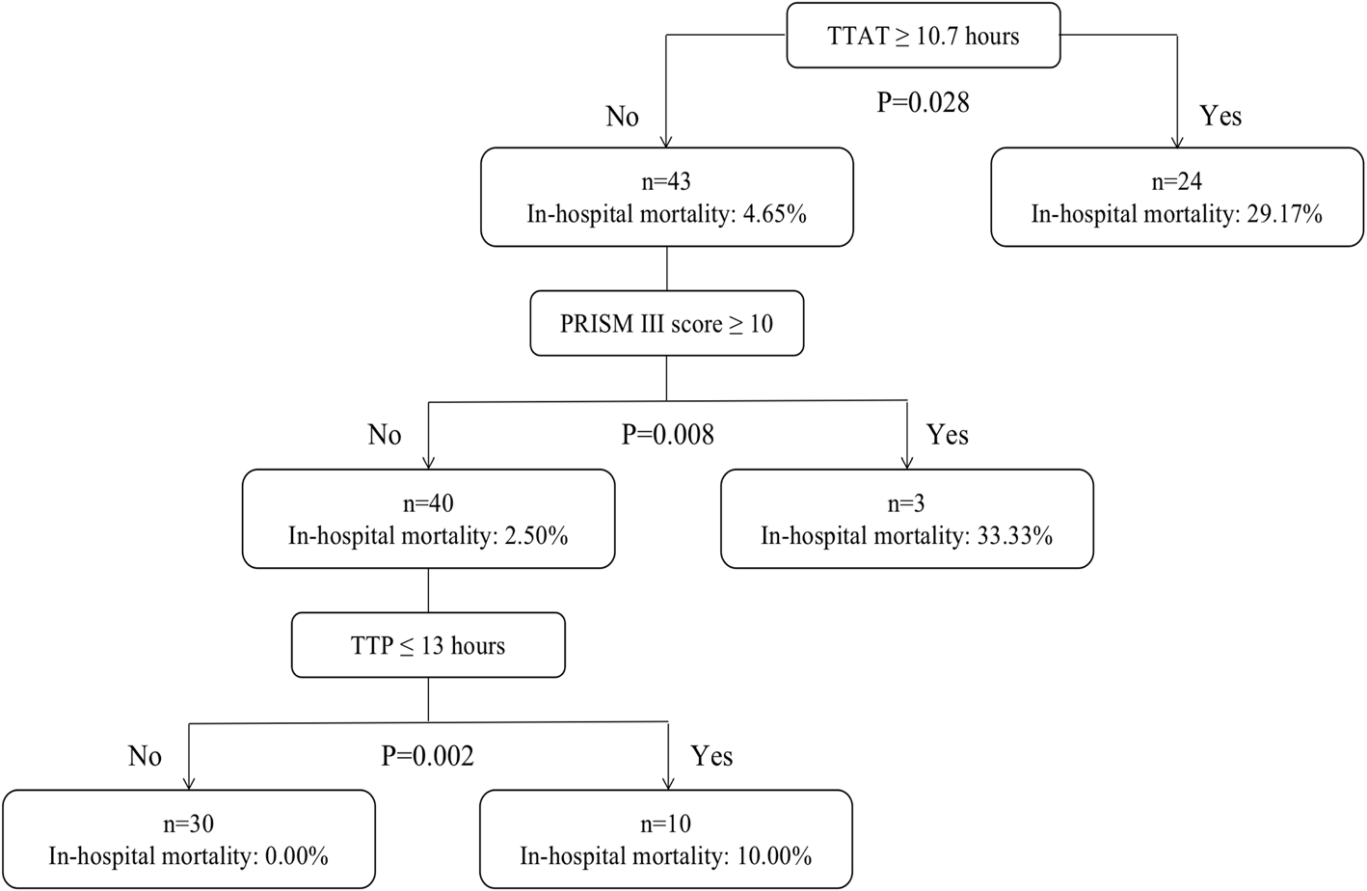
Classification and regression tree analysis of predictors of in-hospital mortality in children with *K. pneumoniae* bloodstream infection

**Fig. 3:**
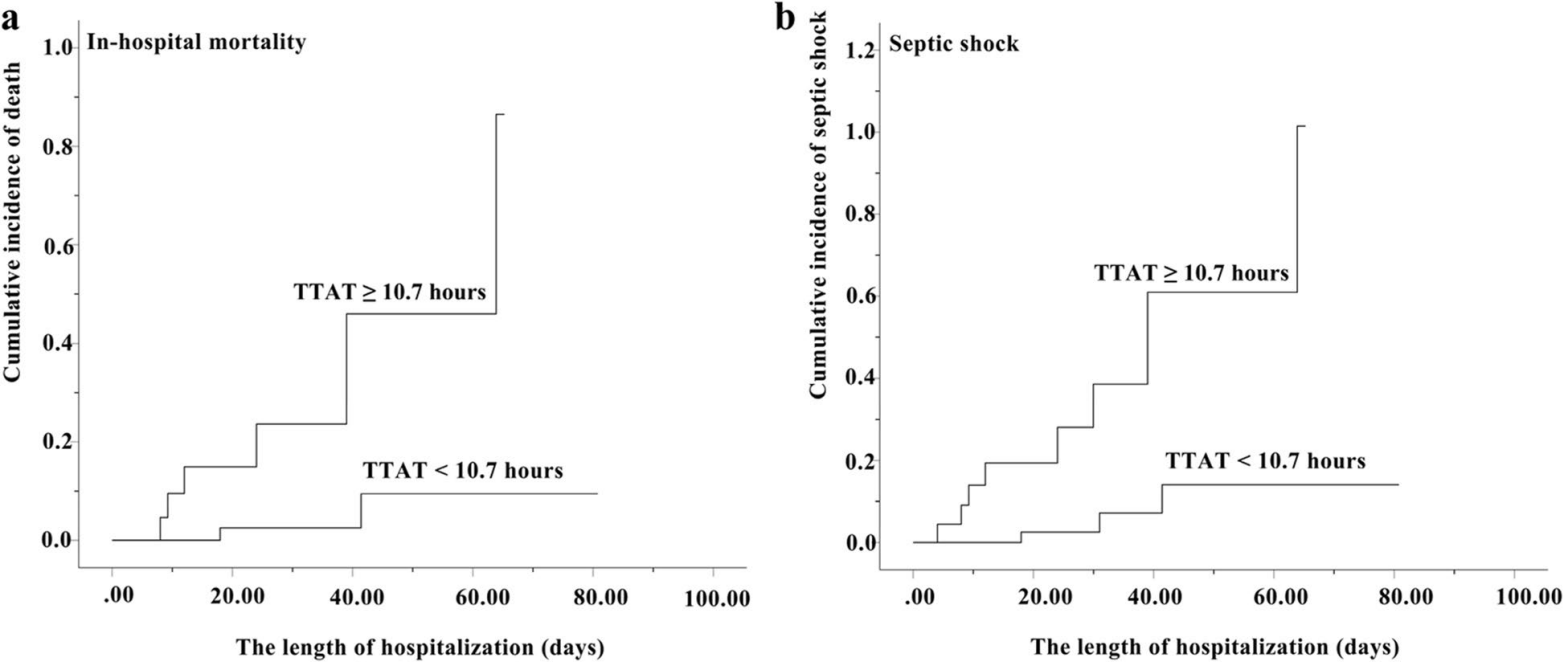
The comparison of patients in different TTAT groups according to in-hospital mortality (**a**) and septic shock (**b**)

**Fig. 4:**
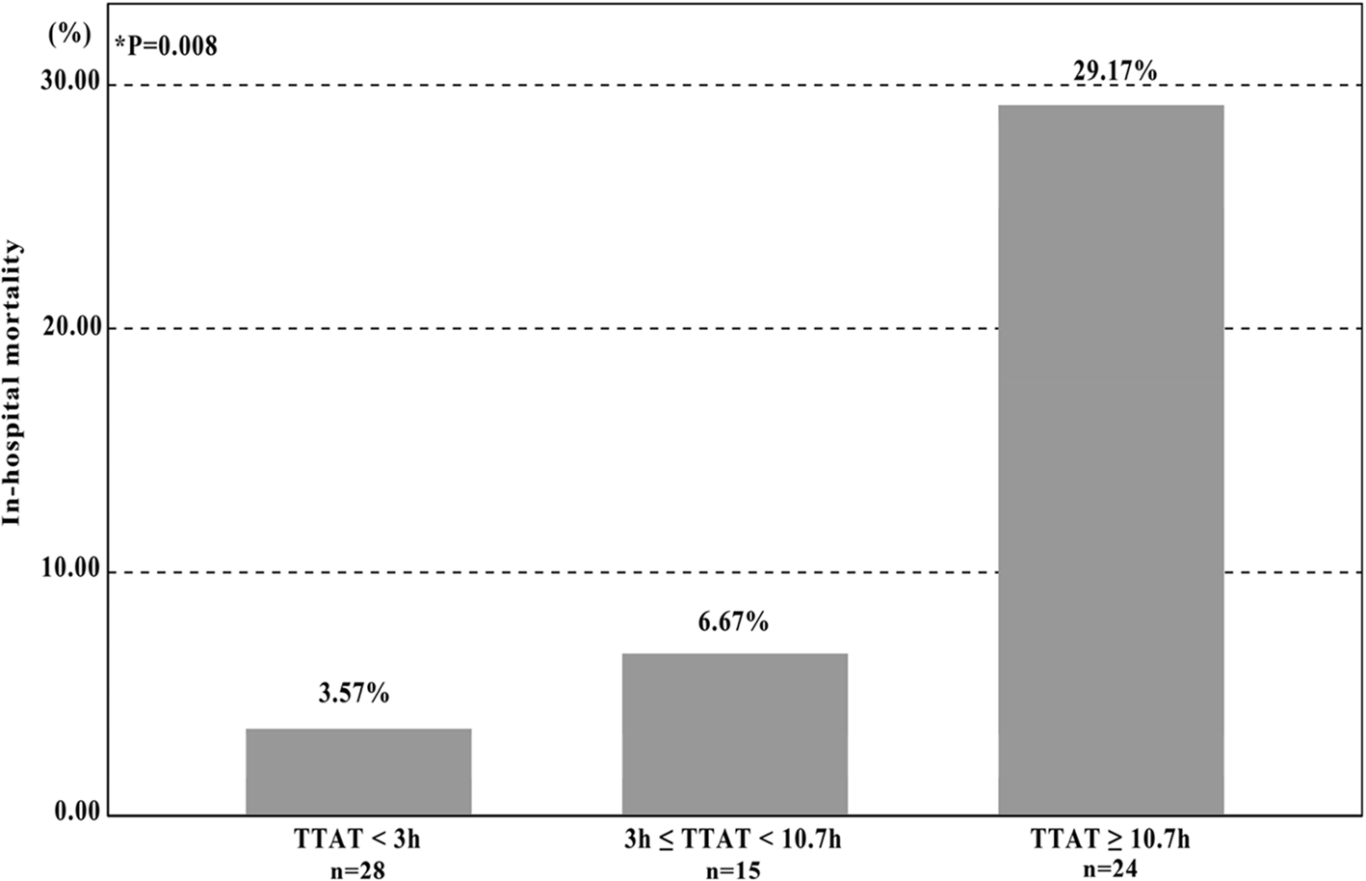
In-hospital mortality stratified by the length of delay in receiving appropriate therapy. *, *P* level for χ^2^ test for linear trend

The TTAT cutoff point derived by CART to delineate the risk of in-hospital mortality was 10.7 h. Patients were classified into early (TTAT < 10.7 h) and delayed therapy group (TTAT ≥ 10.7 h) according to TTAT cutoff point. Twenty-three (34.33%) patients received delayed therapy. Patients received delayed therapy had remarkably higher in-hospital mortality than those received early therapy (29.17% *vs* 4.65%, *P* = 0.028). In patients with TTAT < 10.7 h, higher proportions of PRISM III scores ≥ 10 and TTP ≤ 13 h still significantly indicated higher in-hospital mortality (*P* < 0.01) (Fig. 2). The TTAT cutoff point derived from CART was demonstrated with a good prognostic value in ROC curve analysis (Area under the curve [95% confidence interval (CI)], 0.721 [0.564–0.879], 77.78% sensitivity and 70.69% specificity), with moderate predictive efficacy [24]. Figure 3 showed the Kaplan–Meier survival curve of those patients. In χ^2^ test for a linear trend, patients in TTAT ≥ 10.7 h group had the highest in-hospital mortality when compared to those in TTAT < 3 h and 3 h ≤ TTAT < 10.7 h periods groups. (*P* = 0.008) (Fig. 4).

### Clinical characteristics differences between the early and delayed therapy groups

Table 2 showed the characteristic differences of patients in different TTAT group. When compared with the delayed therapy (TTAT ≥ 10.7 h) group, more patients in early therapy (TTAT < 10.7 h) group had hematologic malignancy (84.09% *vs* 30.43%, *P* < 0.001) and immunosuppression (72.73% *vs* 39.13%, *P* = 0.007). Portion of patients who administrated with carbapenem empirically before the susceptibility tests in early therapy group were prominently higher than that in delayed therapy group (68.18% *vs* 34.78%, *P* = 0.009). Meanwhile, patients received delayed therapy may attribute to the notably higher proportion of empirical third-generation cephalosporin therapy (26.09% *vs* 4.55%, *P* = 0.029) and cephalosporin resistant isolates (39.13% *vs* 13.64%, *P* = 0.017) than those received early therapy. Accordingly, patients received delayed therapy had significantly higher incidence of secondary hypoalbuminemia (56.52% *vs* 20.45%, *P* = 0.002) and septic shock (39.13% *vs* 6.82%, *P* = 0.003), higher proportion of requiring invasive mechanical ventilation (34.78% *vs* 6.82%, *P* = 0.010), higher in-hospital mortality (30.43% *vs* 4.55%, *P* = 0.010) than those early therapy patients. While, the early and delayed therapy groups had no differences with the PRISM III scores, the length of stay before the onset of bloodstream infection and length of the whole hospitalization stay.

**Table 2:** Comparison of clinical characteristics in 67 nosocomial *K. pneumoniae* bloodstream infection children between early therapy and delayed therapy groups

Characteristics	delayed therapy (*n* = 23)	early therapy (*n* = 44)	*P*
Demographic characteristics			
Male (n, %)	13 (56.52%)	29 (65.91%)	0.451
Age (median, IQR)	0.85 (0.52–9.75)	5.75 (2.50–11.05)	0.070
Underlying conditions			
Hematologic malignancy (n, %)	7 (30.43%)	37 (84.09%)	0.000*
Immunosuppression (n, %)	9 (39.13%)	32 (72.73%)	0.007*
Congenital heart disease (n, %)	8 (34.78%)	6 (13.64%)	0.088
Sources of infection			
Respiratory tract (n, %)	11 (47.83%)	26 (59.09%)	0.379
Gastrointestinal tract (n, %)	5 (21.74%)	9 (20.45%)	1.000
Unknown source (n, %)	5 (21.74%)	5 (11.36%)	0.441
Invasive operation (n, %)	2 (8.70%)	3 (6.82%)	1.000
Urinary tract (n, %)	0 (0.00%)	1 (2.27%)	1.000
Drug resistant bacteria phenotypes			
Sulbactam resistant (n, %)	16 (69.57%)	24 (54.55%)	0.234
Extended spectrum beta-lactamase (n, %)	14 (60.87%)	18 (40.91%)	0.120
Cephalosporin resistant (n, %)	9 (39.13%)	6 (13.64%)	0.017*
Tazobactam resistant (n, %)	6 (26.09%)	8 (18.18%)	0.660
Carbapenem resistant (n, %)	3 (13.04%)	4 (9.09%)	0.935
Multidrug resistant (n, %)	2 (8.70%)	4 (9.09%)	1.000
Aminoglycoside resistant (n, %)	2 (8.70%)	2 (4.55%)	0.890
Empiric antimicrobial treatment (n, %)			
Carbapenem (n, %)	8 (34.78%)	30 (68.18%)	0.009*
Fourth-generation cephalosporin (n, %)	3 (13.04%)	6 (13.64%)	1.000
Third-generation cephalosporin (n, %)	6 (26.09%)	2 (4.55%)	0.029*
Tazobactam (n, %)	4 (17.39%)	3 (6.82%)	0.356
Second-generation cephalosporin (n, %)	0 (0.00%)	3 (6.82%)	0.510
Sulbactam (n, %)	2 (8.70%)	0 (0.00%)	0.114
Length of stay before the onset of bloodstream infection (median, IQR)	11.75 (7.14–23.13)	14.42 (10.50–17.19)	0.561
Length of hospitalization stay (median, IQR)	24.00 (12.92–38.88)	30.90 (22.98–46.93)	0.080
The peak of temperature (median, IQR)	39.6 (39.1–40.0)	39.9 (39.3–40.4)	0.135
Antimicrobials administrated prior to blood culture (n, %)	14 (60.87%)	14 (31.82%)	0.022*
With secondary hypoalbuminemia during hospitalization (n, %)	13 (56.52%)	9 (20.45%)	0.002*
PRISM III scores ≥ 10 (n, %)	3 (13.04%)	3 (6.82%)	0.692
TTP ≤ 13 h (n, %)	7 (30.43%)	12 (27.27%)	0.785
Need for invasive mechanical ventilation (n, %)	8 (34.78%)	3 (6.82%)	0.010*
Septic shock (n, %)	9 (39.13%)	3 (6.82%)	0.003*
In-hospital mortality (n, %)	7 (30.43%)	2 (4.55%)	0.010*

*Abbreviations: IQR *inter-quartile range, *PRISM *pediatric risk of mortality, *TTAT *time to appropriate therapy, *TTP* time to positivity

^*^Statistical significance, *P* < 0.05

### Comparisons of clinical characteristics between the survival and non-survival groups

Table 3 compared the clinical characteristics of the survival and non-survival patients. Patients in non-survival group had significantly higher proportions of cephalosporin resistant and extended spectrum beta-lactamase (ESBL) positive isolates, PRISM III scores ≥ 10, TTP ≤ 13 h and TTAT ≥ 10.7 h, higher incidence of requiring invasive mechanical ventilation and septic shock when compared to those in survival group. (*P* < 0.05). The whole hospitalization days and hospitalization days before the onset of bloodstream infection were not prominently associated with outcomes.

**Table 3:** Comparison of clinical characteristics in survival and non-survival groups in 67 nosocomial *K. pneumoniae* bloodstream infection children

Characteristics	Non-survival (*n* = 9)	Survival (*n* = 58)	*P*
Demographic characteristics			
Male (n, %)	4 (44.44%)	38 (65.52%)	0.398
Age (median, IQR)	9.75 (1.72–12.13)	4.29 (0.73–9.69)	0.316
Underlying conditions			
Hematologic malignancy (n, %)	5 (55.56%)	39 (67.24%)	0.757
Immunosuppression (n, %)	5 (55.56%)	36 (62.07%)	0.996
Congenital heart disease (n, %)	1 (11.11%)	13 (22.41%)	0.737
Sources of infection			
Respiratory tract (n, %)	5 (55.56%)	32 (55.17%)	1.000
Gastrointestinal tract (n, %)	2 (22.22%)	12 (20.69%)	1.000
Unknown source (n, %)	2 (22.22%)	8 (13.79%)	0.875
Invasive operation (n, %)	0 (0.00%)	5 (8.62%)	1.000
Urinary tract (n, %)	0 (0.00%)	1 (1.72%)	1.000
Drug resistant bacteria phenotypes			
Sulbactam resistant (n, %)	8 (88.89%)	32 (55.17%)	0.120
Extended spectrum beta-lactamase (n, %)	8 (88.89%)	24 (41.38%)	0.022*
Cephalosporin resistant (n, %)	5 (55.56%)	10 (17.24%)	0.033*
Tazobactam resistant (n, %)	3 (33.33%)	11 (18.97%)	0.585
Carbapenem resistant (n, %)	2 (22.22%)	5 (8.62%)	0.235
Multidrug resistant (n, %)	2 (22.22%)	4 (6.90%)	0.181
Aminoglycoside resistant (n, %)	2 (22.22%)	2 (3.45%)	0.084
Empiric antimicrobial treatment			
Carbapenem (n, %)	6 (66.67%)	32 (55.17%)	0.775
Fourth-generation cephalosporin (n, %)	0 (0.00%)	9 (15.52%)	0.456
Third-generation cephalosporin (n, %)	1 (11.11%)	7 (12.07%)	1.000
Tazobactam (n, %)	1 (11.11%)	6 (10.34%)	1.000
Second-generation cephalosporin (n, %)	0 (0.00%)	3 (5.17%)	1.000
Sulbactam (n, %)	1 (11.11%)	1 (1.72%)	0.252
Length of stay before the onset of bloodstream infection (median, IQR)	16.76 (8.88–33.00)	13.23 (8.47–17.28)	0.211
Length of hospitalization stay (median, IQR)	24.00 (10.63–52.65)	29.46 (22.59–43.74)	0.594
The peak of temperature (median, IQR)	39.6 (39.0–40.0)	39.8 (39.3–40.2)	0.407
Antimicrobials administrated prior to blood culture (n, %)	8 (88.89%)	20 (34.48%)	0.007*
With secondary hypoalbuminemia during hospitalization (n, %)	6 (66.67%)	16 (27.59%)	0.052
PRISM III scores ≥ 10 (n, %)	3 (33.33%)	3 (5.17%)	0.028*
TTP ≤ 13 h (n, %)	6 (66.67%)	13 (22.41%)	0.019*
TTAT ≥ 10.7 h (n, %)	7 (77.78%)	16 (27.59%)	0.010*
Need for invasive mechanical ventilation (n, %)	5 (55.56%)	6 (10.34%)	0.003*
Septic shock (n, %)	9 (100.00%)	3 (5.17%)	0.000*

*Abbreviations*: *IQR* inter-quartile range, *PRISM* pediatric risk of mortality, *TTAT* time to appropriate therapy, *TTP* time to positivity

^*^Statistical significance, *P* < 0.05

### Risk factors of in-hospital mortality

Risk factors of in-hospital mortality were examined by logistic regression analysis. All results were shown in Table 4. Univariate analysis demonstrated that there was positive correlation between in-hospital mortality and the portion of patients with PRISM III scores ≥ 10. So as the patients with early TTP (TTP ≤ 13 h), delayed therapy (TTAT ≥ 10.7 h), requiring for invasive mechanical ventilation, with secondary hypoalbuminemia during hospitalization, ESBL positive isolates, and cephalosporin resistant isolates. According to the multivariate analysis, PRISM III scores ≥ 10 (OR 40.06, 95% CI 2.32–691.35, *P* = 0.011), early TTP (OR 22.60, 95% CI 1.78–287.27, *P* = 0.016), delayed therapy (OR 22.19, 95% CI 1.25–393.94, *P* = 0.035), and need for invasive mechanical ventilation (OR 12.21, 95% CI 1.06–140.67, *P* = 0.045) were independent risk factors of in-hospital mortality.

**Table 4:** Logistic regression analysis of risk factors of in-hospital mortality among 67 K*. pneumoniae* bloodstream infection children

Variables	Univariate analysis			Multivariate analysis		
	OR	95%CI	*P*	OR	95%CI	*P*
PRISM III scores ≥ 10	9.17	1.50–55.93	0.016*	40.06	2.32–691.35	0.011*
TTP ≤ 13 h	6.92	1.52–31.56	0.012*	22.60	1.78–287.27	0.016*
TTAT ≥ 10.7 h	9.19	1.72–48.98	0.009*	22.19	1.25–393.94	0.035*
Need for invasive mechanical ventilation	10.83	2.27–51.71	0.003*	12.21	1.06–140.67	0.045*
Extended spectrum beta-lactamase bacteria	11.33	1.33–96.67	0.026*			
Cephalosporin resistant bacteria	6.00	1.37–26.38	0.018*			
With secondary hypoalbuminemia during hospitalization	3.73	1.03–13.59	0.046*			

*Abbreviations*: *PRISM* pediatric risk of mortality, *TTAT* time to appropriate therapy, *TTP* time to positivity

^*^Indicates statistical significance, *P* < 0.05

### Risk factors of septic shock

Table 5 showed the logistic regression analysis of risk factors of septic shock. In univariate analysis, patients with PRISM III scores ≥ 10, early TTP (TTP ≤ 13 h), delayed therapy (TTAT ≥ 10.7 h), requiring for invasive mechanical ventilation, with ESBL positive isolates and secondary hypoalbuminemia after admission were remarkably associated with the incidence of septic shock. Multivariate analysis demonstrated that delayed therapy (OR 9.87, 95% CI 1.46–66.59, *P* = 0.019), PRISM III scores ≥ 10 (OR 9.69, 95% CI 1.15–81.39, *P* = 0.036), early TTP (OR 8.28, 95% CI 1.37–50.10, *P* = 0.021) and need for invasive mechanical ventilation (OR 6.52, 95% CI 1.08–39.51, *P* = 0.042) were independent risk factors of septic shock.

**Table 5:** Logistic regression analysis of risk factors of septic shock among 67 nosocomial *K. pneumoniae* bloodstream infection children

Variables	Univariate analysis			Multivariate analysis		
	OR	95%CI	*P*	OR	95%CI	*P*
TTAT ≥ 10.7 h	8.79	2.08–37.11	0.003*	9.87	1.46–66.59	0.019*
PRISM III scores ≥ 10	5.78	1.00–33.24	0.049*	9.69	1.15–81.39	0.036*
TTP ≤ 13 h	5.02	1.35–18.67	0.016*	8.28	1.37–50.10	0.021*
Need for invasive mechanical ventilation	10.00	2.33–42.97	0.002*	6.52	1.08–39.51	0.042*
With secondary hypoalbuminemia during hospitalization	5.25	1.17–23.55	0.030*			
Extended spectrum beta-lactamase bacteria	4.17	1.02–17.13	0.047*			
Cephalosporin resistant bacteria	3.21	0.84–12.23	0.087			

*Abbreviations*: *PRISM* pediatric risk of mortality, *TTAT* time to appropriate therapy, *TTP* time to positivity

^*^ indicates statistical significance, *P* < 0.05

## Discussion

In this study, we demonstrated that patients with PRISM III scores ≥ 10, TTP ≤ 13 h, requiring for invasive mechanical ventilation were independently associated with poor outcomes, which were consistent with our previous study [18]. Furthermore, we also showed that delayed therapy (TTAT ≥ 10.7 h) may predict higher incidence of septic shock or in-hospital mortality, which was similar to previous studies indicating delayed appropriate antimicrobial therapy was correlated to poor outcomes [2]. Falcone et al. [9] indicated that appropriate antimicrobial therapy should begin within 24 h from the collection of blood culture in adult carbapenemase-producing *K. pneumoniae* bloodstream infection patients. In this study, we found TTAT ≥ 10.7 h increased 22.19-fold risk of in-hospital mortality and 9.87-fold risk of septic shock in nosocomial *K. pneumoniae* bloodstream infection children. The differences of TTAT thresholds between us and Falcone et al. [9] may be as follows. First, we used different definition of the start point of TTAT. It is more accurate to define the start point of TTAT as onset of bloodstream infection. Obtaining the accurate TTAT for community-acquired infection patients seems to be unrealistic, whereas it`s feasible to gain the information of onset of bloodstream infection and accurate TTAT for nosocomial infection patients. Second, we used CART analysis to find the optimal TTAT cutoff point (10.7 h), and linear χ^2^ test and ROC curve analysis and were also applied to demonstrated it. However, Falcone et al. [9] didn’t explore the optimal TTAT cutoff point. Third, although we both enrolled patients with *K. pneumoniae* bloodstream infection, we concerned patients in different age groups. Two studies [6, 25] stated that TTAT > 3 h indicated higher mortality. Nevertheless, our TTAT was much longer. The explanations may as the following. First, patients with septic shock should administrate appropriate antimicrobials more aggressively than those with sepsis-associated organ dysfunction but without shock [19]. There were 17.91% (12/67) patients with septic shock in our study. While, there were 78.13% (125/160) and 79.23% (103/130) patients with septic shock in Han’s [6] study and Weiss’s [25] study, respectively. The lower proportion of septic shock patients in our study may explain the longer TTAT cutoff point. Second, the methods of defining TTAT cutoff points were different. We used the CART analysis while the other two studies used multivariate analysis.

We found that the secondary hypoalbuminemia during hospitalization may be associated with delayed appropriate antimicrobial therapy. Untimely antimicrobial therapy could lead to persistent bloodstream infection, which leads to increased breakdown and loss of albumin [26]. Low albumin levels may indicate severe condition and poor outcomes [26]. Moreover, this study showed patients received delayed therapy were with significantly higher proportion of empiric third-generation cephalosporin administration before blood culture than those received early therapy. The explanation may as the following. The third-generation cephalosporin is one of the most recommended empiric antimicrobial therapies in nosocomial infections [27]. However, with increased of third-generation resistant *K. pneumoniae* isolates [1], empirical third-generation cephalosporin administration may result in delayed appropriate antimicrobial therapy. *K. pneumoniae* is the most common antimicrobial resistant bacteria [1], and the nosocomial gram-negative bacteria bloodstream infection patients had higher proportion of inappropriate antimicrobial therapy [28]. Therefore, it is very important for clinicians to evaluate whether the empiric antimicrobial therapy is appropriate or not. More than half (38/67, 56.72%) of patients in our study empirically administrated with carbapenem. And the prevalence of carbapenem-resistant *K. pneumoniae* in this study (7/67, 10.45%) was higher than that reported in the European Centre for Disease Prevention and Control (website: http://atlas.ecdc.europa.eu/public/index.aspx?Instance=GeneralAtlas). We consumed that frequently using carbapenem may contribute to carbapenem-resistant *K. pneumoniae* isolate.

Appropriate antimicrobial therapy can improve the clinical outcomes in children with severe bloodstream infection. However, to avoid overtreatment, early recognition of the bloodstream infection and the pathogen is a new challenge to clinicians. In high-income countries, some rapid diagnostic testing technologies can help the clinician to identify *K. pneumoniae* quickly. However, in some low-income countries, the clinical experiences and education level of recognizing *K. pneumoniae* bloodstream infection may be more important. Furthermore, building susceptibility databases of *K. pneumoniae* isolates may help guiding clinicians to choose more appropriate and timely empiric antimicrobial therapy.

This study has some limitations. Firstly, this is a single-center retrospective study, and the sample is relatively small, so that more studies are expected to strength our results. Secondly, we only enrolled patients with nosocomial *K. pneumoniae* bloodstream infection, and this may influence the extrapolation of our data to other populations. Thirdly, when applied our results to clinical practice, we should pay attention to the difference of definitions of the start point of TTAT between us and other studies.

## Conclusions

TTAT could be a prognostic factor in children with nosocomial *K. pneumoniae* bloodstream infection and the timely antimicrobial therapy can improve prognosis. The clinicians should initiate appropriate antimicrobials within 10.7 h of the onset of the *K. pneumoniae* bloodstream infection.

## Data Availability

The datasets used and/or analyzed during the current study available from the corresponding author on reasonable request.

## Abbreviations

CART: Classification and Regression Tree
CI: Confidence interval
CLSI: The Clinical and Laboratory Standards Institute
ESBLs: Extended spectrum beta-lactamases
IQR: Inter-quartile range
MDR: Multi-drug resistant
OR: Odds ratio
PRISM: Pediatric risk of mortality
ROC: The receiver operating characteristic
TTAT: Time to appropriate therapy
TTP: Time to positivity
